# Relationship satisfaction and metabolic health parameters: a cross-sectional study in Burkinabe population of older adults

**DOI:** 10.1186/s12889-024-17998-w

**Published:** 2024-03-15

**Authors:** Adi Lukas Kurniawan, Julius Schretzmann, Rathi Paramastri, Alyssa Cho, Ali Sié, Melanie S. Fischer, Till Bärnighausen, Beate Ditzen

**Affiliations:** 1 Heidelberg Institute of Global Health, Heidelberg University Hospital, Heidelberg University, Heidelberg, Germany; 2 Institute of Medical Psychology, Medical Faculty and University Hospital, Heidelberg University, Heidelberg, Germany; 3 School of Nutrition and Health Sciences, College of Nutrition, Taipei Medical University, Taipei, Taiwan; 4 Epidemiology, Public Health, and Impact, International Vaccine Institute, Seoul, South Korea; 5 Centre de Recherche en Santé de Nouna, Nouna, Burkina Faso; 6 Department of Psychology, University of Marburg, Marburg, Germany; 7 Africa Health Research Institute (AHRI), KwaZulu-Natal, Somkhele, South Africa

**Keywords:** Burkina Faso, Relationship satisfaction, BMI, Waist circumference, HbA1c

## Abstract

**Background:**

Over- and undernutrition coexist in many African countries and pose a threat to metabolic health. This study assessed the associations between relationship satisfaction and Body Mass Index (BMI), waist circumference (WC), and glycated hemoglobin (HbA1c), in a rural population of older adults in Burkina Faso. It also explored potential gender differences and the mediating role of depressive symptoms.

**Methods:**

Data from the “Centre de Recherche en Santé de Nouna (CRSN) Heidelberg Aging Study (CHAS),” a cross-sectional population-based study conducted in 2018 in Burkina Faso, were used in our study. Hierarchical linear regression models were applied for each of the three outcome variables. Among 2291 participants aged 40 years or older who provided data on relationship satisfaction, 2221, 2223, and 2145 participants had BMI, waist circumference (WC), and HbA1c values respectively.

**Results:**

Higher relationship satisfaction (CSI-4 score) was associated with increased BMI (β = 0.05, *p* = 0.031) and WC (β = 0.12, *p* = 0.039). However, the association of CSI-4 and BMI became non-significant after controlling for depressive symptoms (PHQ-9 score) and physical inactivity (BMI: β = 0.04, *p* = 0.073). Depressive symptoms fully mediated the relationship between relationship satisfaction and BMI (β = -0.07, *p* = 0.005). There was no significant association between relationship satisfaction and HbA1c. These results were consistent across genders and age groups.

**Conclusion:**

Higher relationship satisfaction may lead to increased body weight among Burkinabe adults aged 40 years and older, and depressive symptoms may be a mediator in this association.

**Supplementary Information:**

The online version contains supplementary material available at 10.1186/s12889-024-17998-w.

## Introduction

Non-communicable diseases are rapidly rising in Sub-Saharan Africa (SSA) [1], including increasing obesity rates and the threat of double-burden malnutrition [2, 3]. The region experienced an absolute rise of 1.14 kg/m^2^ and 1.37 kg/m^2^ in men and women between 1985 and 2017 [4]. A change in diet combined with increased urbanization and a more sedentary lifestyle are considered to be driving factors in these developments [3, 5]. Notably, besides traditional behavioral health risks, there is an increasing interest in how the quality of social relationships might more directly affect (metabolic) health [6–8].

Social and romantic relationships positively impact health, leading to longer and healthier lives [9, 10]. A satisfied relationship is seen as health-promoting as it may provide a sense of control, belonging, meaning, and security while stimulating healthy and discouraging unhealthy behavior [9, 11]. Thus, the quality of relationships, including communication and positivity, plays a significant role in determining health outcomes, surpassing structural criteria such as marital status, or living arrangements [9]. Particularly, studies have demonstrated that strong and fulfilling relationships can significantly mitigate the adverse impacts of stress on health, whereas low-quality relationships can act as stressors and have detrimental effects on well-being [12, 13]. Additionally, several studies have explored the connection between depressive symptoms, psychosocial stress, and metabolic health, revealing the co-occurrence of depressive symptoms and perceived stress is generally associated with poorer metabolic health outcomes and increased inflammatory markers [14–16]. However, only a limited number of studies have shown associations between romantic relationships and metabolic outcomes, including weight gain and diabetes prevalence, with gender-specific results. For instance, a longitudinal study conducted in the US found marital quality– and especially marital support– was inversely associated with weight gain after a period of 10 years and these results were evident only in men [8]. Similarly, a cross-sectional population-based study in the US also discovered that higher relationship satisfaction was associated with decreased odds of having diabetes in men only [17].

It is important to note that these findings are predominantly from high-income countries, and it is likely that the association may vary across different cultures. Furthermore, the applicability of these findings to low- and middle-income countries remains unclear due to limited research exploring the link between relationship satisfaction and metabolic health in these regions. Burkina Faso, like other Sub-Saharan African (SSA) countries, faces metabolic health problems. Data from a nationally representative survey in 2013 revealed that the prevalence of overweight and obesity was 13.82% and 4.84%, respectively [18]. Moreover, the same survey indicated a 9% prevalence of abnormal glucose regulation and a 5.8% prevalence of diabetes in Burkina Faso [19]. However, to the best of our knowledge, there has been limited study investigating the association between relationship satisfaction and metabolic health parameters in SSA countries. Therefore, this study aimed to investigate the association between relationship satisfaction and BMI, waist circumference (WC), and glycated hemoglobin (HbA1c) in a population-based sample from Burkina Faso. We hypothesize that individuals with higher levels of relationship satisfaction will exhibit lower values of BMI, WC, and HbA1c. Additionally, this study seeks to examine potential gender differences in these associations.

## Methods

### Study sample and data collection

**Fig. 1:**
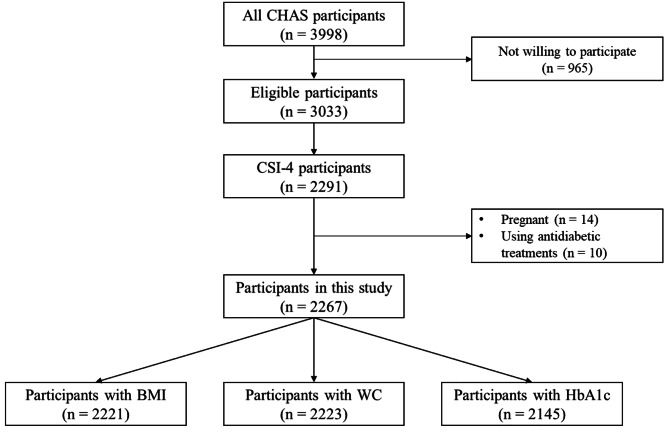
Flowchart number of participants included in this study. BMI: Body Mass Index; CHAS: Centre de Recherche en Santé de Nouna Heidelberg Aging Study; CSI: Couples Satisfaction Index 4; WC: waist circumference

The data analyzed in this study were obtained from the “Centre de Recherche en Santé de Nouna (CRSN) Heidelberg Aging Study (CHAS),” which aimed to assess the health status of older adults in rural Burkina Faso. The study population was randomly selected from the Nouna Health and Demographic Surveillance System (HDSS), covering both 7 sectors of semi-urban and 58 rural villages with a diverse population at Nouna town, North-Western Burkina Faso. The baseline study was conducted in 2018 and a detailed description of the sampling process has been published elsewhere [20]. From 3998 participants, a total of 2291 participants in a relationship provided data on relationship satisfaction. After applying inclusion criteria for being 40 years or older, a primary resident of the household for at least the preceding six months, being willing and able to give informed consent, not being pregnant, not using antidiabetic treatment, and having no missing value in the outcome variables, the final analysis included 2221 participants for BMI, 2223 for WC, and 2145 for HbA1c (Fig. 1).

### Data measurements

Data for this study was collected through interviews conducted at respondents’ homes by trained fieldworkers. The interviews took place between May and July 2018 and were conducted in various languages (Dioula, French, Mooré, and other native languages). The investigation involved questionnaires, measurements of physical, cognitive, and psychosocial functioning, physical measurements such as weight and height, and a blood draw for laboratory analysis. Anthropometric measurements were taken twice and averaged. The study focused on two dimensions of metabolic health: body weight, measured by BMI and waist circumference (WC), and glucose metabolism, measured by glycated hemoglobin HbA1c. BMI values were calculated as the mean body weight divided by the squared mean height. Waist circumference values were calculated as the average value of the two measurements being done for each participant (unit: cm). HbA1c values were analyzed using a Jactron Pictus 400 machine. We use the percentage of HbA1c in total hemoglobin as a unit of measurement.

Relationship satisfaction was assessed using the French translated Couples Satisfaction Index 4 (CSI-4) [21]. It consists of four items including: please indicate the degree of happiness in your relationship, I have a warm and comfortable relationship with my partner, how rewarding is your relationship with your partner, and in general, how satisfied are you with your relationship. Responses were captured with a scale ranging from 0 (“Extremely Unhappy” for item 1 or “Not at all true” for items 2–4) to 5 (“Extremely Happy” for item 1 or “Completely true” for item 2–4). The original CSI-4 has an additional response option (“6– Perfect”) for the first item, thus scores range from 0 to 21, and scores below 13.5 indicate notably dissatisfied relationships [21]. However, for conformity reasons and to facilitate administration via interview, the study team decided to use the same point range for all items, thus omitting the “6– Perfect” option of item 1. Therefore, CSI-4 scores for this study range from 0 to 20. In the current study, the CSI-4 yielded good internal consistency (Cronbach’s α = 0.84) with an approximately symmetric normal distribution, indicating that this instrument is capable of discerning differences in relationship satisfaction in this Burkinabe population.

### Other covariates

Basic demographic information (age, gender, and 6 categories of ethnicity Dafin, Bwaba, Mossi, Peulh, Samo, and “Other”) was included as covariates. Additional demographic information included education and wealth, which was assessed as years of full-time schooling for education status and using 37 asset ownership indicators and dwelling characteristics such as livestock, bank account, and distance to a water source for wealth status. A wealth index was created based on principal component analysis as described in the previous study [22], and the original sample was divided into quintiles of wealth levels.

Depressive symptoms were assessed using the Patient Health Questionnaire 9 (PHQ-9), which consists of nine items measuring the frequency of depressive symptoms over the past 2 weeks [23]. The answers range from “0– Not at all” to “3– Nearly every day“ and the scores range from 0 to 27, with higher scores indicating more severe symptoms. The study used a French version of the PHQ-9, which was found to have no significant differences compared to the English version [24].

Physical inactivity was estimated by measuring participants’ total sitting time per week. They were asked to estimate the time spent sitting or reclining on weekdays and weekends, which was then summed up to determine the total sitting time during a week. Moreover, hypertension was defined as either a systolic blood pressure ≥ 140 mm/Hg, or a diastolic blood pressure ≥ 90 mm/Hg or receiving antihypertensive treatment [25]. An interaction term between gender and CSI-4 was included to investigate the moderating effect of gender on the association between relationship satisfaction and outcomes. Furthermore, although information on relationship duration was not available, it is important to note that this variable is typically correlated with age [26]. Therefore, to address this potential modification effect, an interaction term for age and CSI-4 was included as well in the analysis.

### Data analysis

The statistical analyses were performed using STATA v.16 (StataCorp LP, TX, USA). Descriptive data were presented as the median and interquartile range for continuous variables, and count and percentage values for categorical variables. Meanwhile, group differences were assessed using Kruskal-Wallis test for continuous variables and Pearson’s Chi-squared tests for categorical variables.

Four hierarchical linear regression models using *nestreg: reg* command were applied for each outcome variable. Model 1 adjusted with basic demographic variables (age, gender, and ethnicity). Model 2 added wealth and education to the demographic variables. The HbA1c models included hypertension and BMI in addition to previous variables. Model 3 added PHQ-9 and sitting time. Model 4 included interaction terms between CSI-4:Age and CSI-4:Gender to explore moderating effects. Variables in the interaction terms (CSI-4 and age) were mean-centered for interpretability. Moreover, the mediation analysis of PHQ-9 with CSI-4 on BMI was performed using *sem* and *medsem* commands with bootstrap 500 replications in STATA. Dummy variables were created for each level of a categorical variable. Reference levels were “male” for gender, “Dafin” for ethnicity, and “Q1” for wealth. The beta coefficients, 95% confidence intervals (CIs), R^2^, adjusted R^2^, and *p*-values are reported for each model. A *P*-value of < 0.05 was considered statistically significant.

## Results

### Descriptive characteristics

Table 1 presents gender-stratified summary statistics. The CSI-4 scores differed significantly between men and women, with median scores of 12.5 and 12, respectively (*p* < 0.001). There were also significant differences in BMI scores (median: 21.4 kg/m² for men and 21.7 kg/m² for women; *p* = 0.001) and waist circumference values (median: 82 cm for men, 85 cm for women; *p* < 0.001). However, there was no significant difference in HbA1c values (*p* = 0.063). The median PHQ score was 3, with men scoring 3 and women scoring 4 (*p* < 0.001). The median score falls between the cutoff for minimal and mild depressive symptoms (0 to 5 scores). Moreover, participants spent approximately 21 h per week sitting or reclining, which equates to around 3 h per day.

**Table 1:** Descriptive characteristics of the study sample stratified by gender

	Male	Female	Total	*p*-value
**N**	1368	899	2267	
**Age**				**0.010** ^**2**^
< 60 years old	1069 (78.2)	742 (82.6)	1811 (80.0)	
≥ 60 years old	298 (21.8)	156 (17.4)	454 (20.0)	
**Ethnicity**				0.198^2^
Dafin	560 (40.9)	331 (36.8)	891 (39.3)	
Bwaba	378 (27.6)	285 (31.7)	663 (29.2)	
Mossi	193 (14.1)	113 (12.6)	306 (13.5)	
Peulh	115 (8.4)	86 (9.6)	201 (8.9)	
Samo	97 (7.1)	66 (7.3)	163 (7.2)	
Other	25 (1.9)	18 (2.0)	43 (1.9)	
**Wealth**, ***quintiles***				0.449^2^
1	201 (14.7)	155 (17.3)	356 (15.7)	
2	274 (20.0)	186 (20.7)	460 (20.3)	
3	300 (21.9)	180 (20.0)	480 (21.2)	
4	300 (21.9)	196 (21.8)	495 (21.8)	
5	294 (21.5)	182 (20.2)	476 (21.0)	
**Years of study**				**< 0.001** ^**1**^
0	1060 (77.5)	806 (89.7)	1866 (82.3)	
1–6 (primary)	214 (15.6)	74 (8.2)	288 (12.7)	
7–15 (secondary)	79 (5.8)	18 (2.0)	97 (4.3)	
≥ 16 (tertiary)	15 (1.1)	1 (0.1)	16 (0.7)	
**BMI**, ***kg/m²***	21.4 (4.3)	21.7 (5.7)	21.5 (4.7)	**0.001** ^**1**^
**WC**, ***cm***	82 (11)	85 (14.5)	82.5 (13)	**< 0.001** ^**1**^
**HbA1c**, ***%***	5.4 (0.5)	5.3 (0.5)	5.3 (0.5)	0.063^1^
**CSI-4**	12.5 (5)	12 (5)	12 (5)	**< 0.001** ^**1**^
**PHQ-9**	3 (5)	4 (5)	3 (5)	**< 0.001** ^**1**^
**Sitting**, ***h/week***	21 (14)	21 (14)	21 (14)	0.935^1^

Data are presented as median (interquartile range) for the continuous variables and number (percentage) for categorical variables

BMI: Body Mass Index; CSI-4: Couples Satisfaction Index 4; HbA1c: Glycated Hemoglobin A1c; PHQ-9: Patient Health Questionnaire 9; WC: waist circumference

^1^*P*-value was obtained with Kruskal-Wallis test

^2^*P*-value was obtained with Pearson Chi-Squared test

### Multiple linear regression models

#### Relationship satisfaction and BMI

Our study found that relationship satisfaction was minimally and positively associated with BMI as shown in Table 2. The association remained significant even after adjusting for demographic characteristics. Specifically, a one-unit increase in CSI-4 was associated with a 0.08 kg/m² increase in BMI (β_1_ = 0.08, 95% CI: 0.04–0.13, *p* < 0.001) when adjusting for age, gender, and ethnicity. Further adjustment for wealth and education resulted in a 0.05 kg/m² increase in BMI (95% CI: 0.00–0.09, *p* = 0.031). However, when accounting for depressive symptoms score and physical inactivity (sitting time), the association further weakened and became non-significant (β_3_ = 0.04, 95% CI: -0.00–0.08, *p* = 0.073). In comparison to individuals with a normal BMI, depressive symptoms were associated with increased likelihood of being underweight. Meanwhile, there was no association found between depressive symptoms and being overweight or obese (Supplementary Table 1).

**Table 2:** Hierarchical linear regression models for BMI (*n* = 2221)

Predictors	Model 1				Model 2				Model 3				Model 4		
	β	95% CI	P		β	95% CI	P		β	95% CI	P		β	95% CI	P
CSI-4	0.08	0.04–0.13	< 0.001		0.05	0.00–0.09	0.031		0.04	-0.00–0.08	0.073		0.06	-0.00–0.11	0.054
PHQ-9									-0.06	-0.11– -0.02	0.008		-0.06	-0.11– -0.02	0.009
Sitting h/week									0.01	-0.00–0.02	0.181		0.01	-0.00–0.02	0.180
CSI: Gender													-0.04	-0.13–0.05	0.375
CSI:Age													0.00	-0.00–0.01	0.857
R^2^/adj.R^2^	0.077/0.074				0.162/0.157				0.165/0.159				0.165/0.158		

Model 1: adjusted for age, gender, and ethnicity

Model 2: additionally adjusted for wealth and education

Model 3: additionally adjusted for PHQ-9 and sitting time

Model 4: additionally adjusted for interaction terms between CSI-4 and age/gender

CSI-4: Couples Satisfaction Index 4; PHQ-9: Patient Health Questionnaire 9

**Fig. 2:**
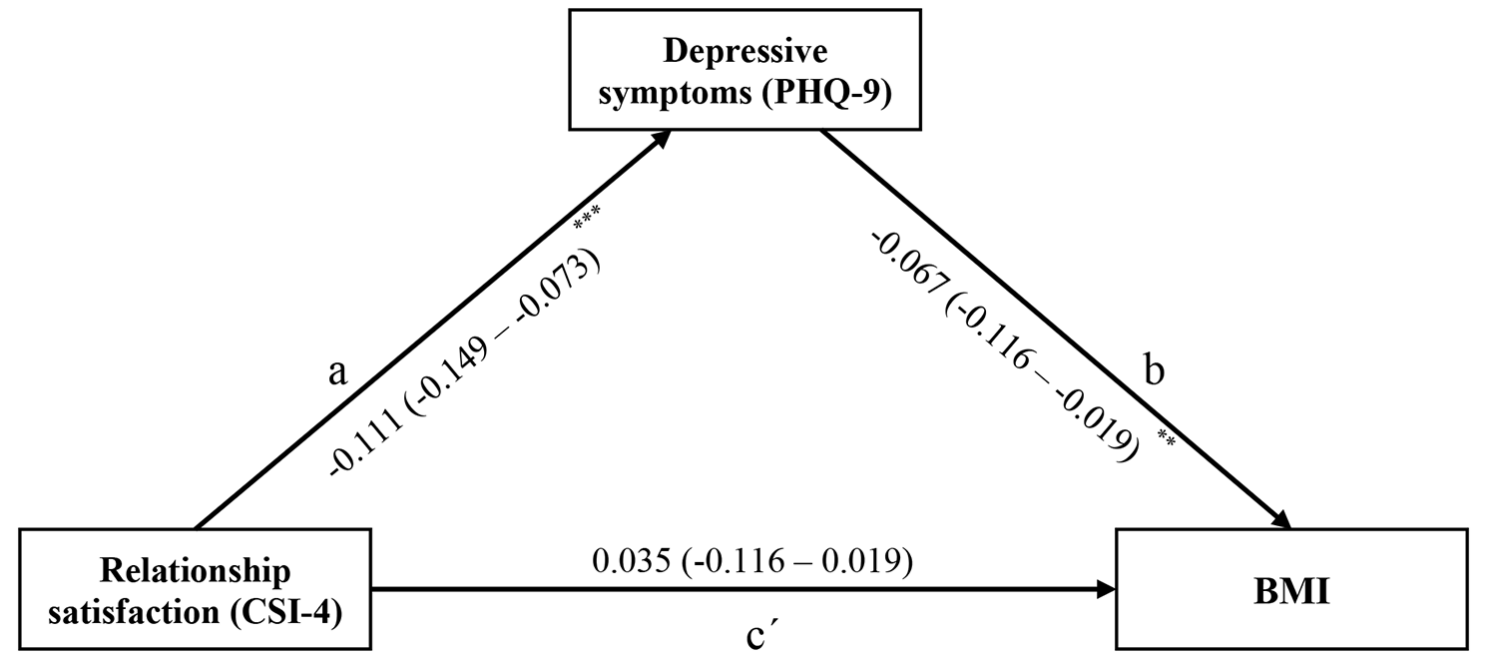
Mediation model of depressive symptoms measured by PHQ-9 on relationship satisfaction measured by CSI-4 and BMI. Data are presented as beta coefficient and 95% Confidence Intervals in parentheses and adjusted for model 4. BMI: Body Mass Index; CSI-4: Couples Satisfaction Index 4; PHQ-9: Patient Health Questionnaire 9. *** *p* < 0.001, ** *p* < 0.01

Additional analyses indicated that the PHQ-9 mediated the relationship between CSI-4 and BMI, as shown in Fig. 2. CSI-4 scores were significantly and negatively associated with PHQ-9 scores (path a; β = -0.111; *p*-value < 0.001). PHQ-9 scores were also significantly associated with BMI values (path b; β = -0.067; *p*-value = 0.005). In the full model, CSI-4 scores were not significantly associated with BMI values (path c’; β = 0.035; *p*-value = 0.102). Using bootstrapping procedures, the significance of the indirect effect of CSI-4 on BMI via PHQ-9 (a*b = -0.111*-0.067 = 0.007) was tested. The bootstrapped unstandardized indirect effect was 0.007, and the 95% confidence interval ranged from 0.002 to 0.013. Thus, the indirect effect was statistically significant (*p*-value = 0.014). The Monte Carlo mediation analysis test obtained with *medsem* showed significant results (β = 0.008, 95% CI: 0.002–0.014, *p* = 0.011), which suggests that there was full mediation and the ratio of the indirect effect to the total effect (RIT) (0.007 / 0.035 = 0.213) showed that 21% of the effect of CSI-4 on BMI mediated by PHQ-9 scores. Moreover, no evidence was found for a moderating effect of gender or age on the association between relationship satisfaction and BMI, as the interaction terms yielded non-significant results.

#### Relationship satisfaction and waist circumference

Similar to the previous findings, relationship satisfaction was positively associated with WC (Table 3). The association remained significant after adjusting for various demographic characteristics. Specifically, adjustment for Model 2 resulted in a 0.13 cm increase in WC for each one-unit increase in CSI scores (95% CI: 0.02–0.24, *p* = 0.024). Further adjustments for depressive symptoms score and sitting time resulted in a 0.12 cm increase in WC (95% CI: 0.01–0.23, *p* = 0.039).

**Table 3:** Hierarchical linear regression models for WC (*n* = 2223)

Predictors	Model 1				Model 2				Model 3				Model 4		
	β	95% CI	P		β	95% CI	P		β	95% CI	P		β	95% CI	P
CSI-4	0.23	0.11–0.35	< 0.001		0.13	0.02–0.24	0.024		0.12	0.01–0.23	0.039		0.12	-0.02–0.27	0.108
PHQ-9									-0.10	-0.22–0.02	0.119		-0.10	-0.22–0.02	0.118
Sitting h/week									0.01	-0.02–0.04	0.464		0.01	-0.02–0.04	0.468
CSI: Gender													-0.01	-0.23–0.22	0.924
CSI:Age													0.00	-0.01–0.01	0.646
R^2^/adj.R^2^	0.050/0.047				0.155/0.150				0.156/0.150				0.156/0.150		

Model 1: adjusted for age, gender, and ethnicity

Model 2: additionally adjusted for wealth and education

Model 3: additionally adjusted for PHQ-9 and sitting time

Model 4: additionally adjusted for interaction terms between CSI-4 and age/gender

CSI-4: Couples Satisfaction Index 4; PHQ-9: Patient Health Questionnaire 9

Secondary analyses indicated that PHQ-9 did not act as a significant mediator in the association between CSI-4 and WC (Supplementary Fig. 1). Moreover, neither of the interaction terms (gender and age) yielded significant results, suggesting no evidence of a moderating effect on the association between relationship satisfaction and WC.

#### Relationship satisfaction and HbA1c

There was no significant association between relationship satisfaction and HbA1c in any of the models (Table 4). However, significant associations were observed for age, ethnicity, wealth, hypertension, and BMI (Supplemental Table 2). The findings indicate that increased HbA1c levels were associated with age, wealth, hypertension, and higher BMI, while belonging to a certain ethnicity (referred to as Bwaba and Peulh) was linked to lower HbA1c levels. The interaction terms CSI x gender and CSI x age did not significantly affect HbA1c.

**Table 4:** Hierarchical linear regression models for HbA1c (*n* = 2145)

Predictors	Model 1				Model 2				Model 3				Model 4		
	β	95% CI	P		β	95% CI	P		β	95% CI	P		β	95% CI	P
CSI-4	0.00	-0.00–0.01	0.381		-0.00	-0.01–0.01	0.829		-0.00	-0.01–0.01	0.899		0.00	-0.01–0.01	0.590
Hypertension					0.11	0.05–0.15	< 0.001		0.10	0.05–0.15	< 0.001		0.10	0.05–0.15	< 0.001
BMI					0.02	0.02–0.03	< 0.001		0.02	0.02–0.03	< 0.001		0.02	0.02–0.03	< 0.001
PHQ-9									0.00	-0.00–0.01	0.346		0.00	-0.00–0.01	0.337
Sitting h/week									0.00	-0.00–0.00	0.081		0.00	-0.00–0.00	0.079
CSI:Gender													-0.01	-0.02–0.01	0.337
CSI:Age													-0.00	-0.00–0.00	0.837
R^2^/adj.R^2^	0.023/0.019				0.084/0.078				0.086/0.079				0.087/0.078		

Model 1: adjusted for age, gender, and ethnicity

Model 2: additionally adjusted for wealth, education, hypertension, and BMI

Model 3: additionally adjusted for PHQ-9 and sitting time

Model 4: additionally adjusted for interaction terms between CSI-4 and age/gender

β: beta-coefficient; SE: Standard Error; p: *p*-value; CSI-4: Couples Satisfaction Index 4; PHQ-9: Patient Health Questionnaire 9

## Discussion

This study examined the association between relationship satisfaction and metabolic health markers (BMI, WC, and HbA1c) in 2237 adults aged 40 years and older from Burkina Faso. The findings showed that relationship satisfaction was positively associated with body weight (BMI and WC), contrary to expectations. However, depressive symptoms fully mediated the association between relationship satisfaction and BMI. No significant association was found between relationship satisfaction and HbA1c. Gender and age did not moderate the association between relationship satisfaction and metabolic outcomes.

### Relationship satisfaction and body weight

Relationship satisfaction was positively associated with body weight, and significant associations were found after adjustment for various demographic characteristics. Although our study revealed that people with higher BMI tended to be those who were more satisfied in their relationships, it is important to note that the majority of our participants had a BMI within the “normal” range (*n* = 1458, 65.6%). When interpreted from a Western perspective, the positive association found in our study might support the mating market theory, which suggests that weight maintenance is motivated to attract a mate. Thus, according to this theory, individuals in less satisfying marriages may be less likely to gain weight to improve their appearance to be more appealing to potential new partners. Given a very slim ideal body image this suggests that satisfying romantic relationships can undermine weight change concerns [27]. However, caution must be taken when interpreting these results. Firstly, the participants in the previous study were younger and at a different stage of life. Furthermore, the cultural context in Burkina Faso differs significantly, and a Burkinabe spouse may be less likely to seek a divorce. Therefore, the mating market theory may not be applicable in the Burkinabe context.

In contrast to previous studies conducted on Western samples [7, 8, 28, 29], which consistently reported a negative association between relationship satisfaction and body weight, the present study found different findings. However, the association between relationship satisfaction and BMI in this study may not necessarily indicate an unhealthy body weight. A review study about body image perception among Africans reported that preferences for slightly overweight body sizes are common in many African populations, especially those from rural areas [30]. In line with these findings, a study among adolescent girls from the Nouna HDSS found that most of their respondents desired a larger body [31]. Therefore, a higher body weight might represent a beauty or status ideal in the population observed in this study and thus reciprocally improve both partners’ relationship satisfaction. Moreover, within a society where hunger, food insecurity, and limited food availability especially high-calorie and energy-dense foods are common [32], there might be a perception that a higher BMI or larger body size is desirable, as it might correspond to greater access to food, resource, and overall stability. Other metabolic risk factors such as alcohol consumption, tobacco use, a sedentary lifestyle, and access to leisure activities may also play a role in mediating this association. These factors are more readily accessible to individuals in high-income countries compared to Burkina Faso’s population. Future studies could explore this association in populations at higher metabolic risk, urban residents, and individuals with higher socioeconomic status.

Furthermore, our study found that the association between relationship satisfaction and BMI seems to be entirely mediated by depressive symptoms. This result aligns with previous studies indicating a close link between relationship satisfaction and depressive symptoms [33, 34]. This also serves as a validation of the results regarding relationship satisfaction and BMI: In this present African sample, we find a linear and negative association of depressive symptoms with BMI, which is in contrast to previous studies from high-income countries [35]. Being undernourished is a serious health threat to Sub-Saharan residents. However, more refined analyses in our sample do not support curvilinear trend. In comparison to individuals with a normal BMI, depressive symptoms were associated with increased likelihood of being underweight. Conversely, no association was found between depressive symptoms and being overweight or obese (Supplementary Table 1). Interestingly, our study also found no evidence for depressive symptoms mediating the association between relationship satisfaction and waist circumference. This finding contradicts the expected positive association between depressive symptoms and obesity according to prior evidence. Previous studies have consistently reported a positive bidirectional association between depressive symptoms and obesity [36], as well as a positive association between depressive symptoms and metabolic syndrome [37]. However, in our prior analysis of the same sample, we did not observe a significant association between depressive symptoms and metabolic syndrome [38], aligning with findings from other studies conducted among sub-Saharan African populations [39, 40]. The significant mediation of depressive symptoms in the association between relationship satisfaction and BMI indicates a potential sub-clinical association between depressive symptoms and BMI in this sample of Burkinabe adults.

Additionally, the lack of awareness and knowledge about metabolic risk factors may contribute to the observed association. Engaging in a healthy lifestyle requires individuals to recognize unhealthy behaviors and replace them with healthy ones. However, a study has shown a lack of awareness and knowledge about cardiovascular diseases and their risk factors in sub-Saharan African populations [41]. This lack of health literacy may lead couples to live metabolically unhealthy lives, despite their desire to prioritize their health. It is important to note as well that health literacy and, to some extent, cultural value play a role in shaping an individual’s knowledge, attitude, and belief regarding their perception of body image. Future studies should explore the role of (metabolic) health literacy as a potential mediating factor, as it could inform public health interventions.

### Relationship satisfaction and diabetes

Our study found that there was no significant association between relationship satisfaction and HbA1c levels. The models used in the study had limited explanatory power, as they could only account for 7.9% of the variance in HbA1c values. This suggests that the variables used in the models, including relationship satisfaction, were not well-suited to explaining the differences in HbA1c values. While other studies have reported significant associations between relationship quality and diabetic outcomes, the findings have not always been consistent [17, 42, 43].

It is possible that a minimum level of exposure to health-deteriorating factors is necessary to establish an association between relationship satisfaction and HbA1c levels. Additionally, theories on the impacts of low relationship quality on health suggests that stress plays a central role in mediating the association between relationship quality and diabetes. It is possible that specific negative aspects of relationship quality, such as conflict or criticism, have a greater impact on participants’ stress levels than overall relationship satisfaction, which was measured in the present study. Previous studies [17, 40, 41] used a more comprehensive assessment of relationship quality, which may explain why they have found significant associations.

### Relationship satisfaction and gender

Our study also found that women reported lower relationship satisfaction than men, but gender did not have a specific effect on the association between relationship satisfaction and metabolic outcomes. The findings regarding gender effects in previous studies have been inconsistent. Some studies have found gender-specific effects, such as a study in the US that found a link between positive marital quality and lower prevalence of diabetes only in men [17], while another study in New Zealand found that relationship quality was negatively correlated with weight gain only in women [28]. Additionally, a notable US study found that negative marital quality in men and positive marital quality in women were associated with lower odds of incident diabetes [42]. However, several studies did not find gender effects in this area [7, 29, 44]. Women are often suggested to be more emotionally involved in and affected by close or intimate relationships, which potentially leading to a greater influence on their partner’s health and be more sensitive to the quality of their relationship, resulting in more pronounced health effects [45]. Nevertheless, generalizing gender differences in the association between romantic relationships and metabolic health may not be suitable due to significant cultural variations in gender concepts [46]. Moreover, since most studies in this field were conducted amongst populations from high-income countries (HIC), data may not be transferable to populations in low-income countries (LIC). Therefore, research conducted among populations living in low-income countries is a valuable addition to the field.

### Strengths and limitation

The strengths of the study include its large and randomly selected population-based sample, which enhances the validity of the findings for the Nouna region and possibly other regions in Burkina Faso or sub-Saharan Africa. To our knowledge, the study is also the first to investigate relationship satisfaction in a large sub-Saharan population using the CSI-4 [21]. Results suggest that per se the CSI-4, as a highly economic and unidimensional measurement instrument, can give a valid impression of relationship satisfaction in non-Western populations and can therefore be used to assess biopsychological associations in different cultures. Based on this, this study is the first to focus on the association between relationship satisfaction and metabolic health in sub-Saharan Africa. The lower rate of marriage dissolution in Burkina Faso compared to the US [47, 48] provides an opportunity to study the connection between relationship satisfaction and health on a broader scale. In Burkina Faso, women face significant social repercussions after a divorce, making it challenging for them to return to their families and remarry. As a result, couples may stay married for longer, even if the quality of their relationship is poor.

However, our study has several limitations. First, the results may not be generalizable to younger populations as the study only included individuals aged 40 years and older. Second, a cross-sectional study cannot establish causal explanations for the observed associations. Third, the translation of the CSI-4 into French and the local language introduces the risk of translation and interpretation bias, although the instrument showed good internal consistency and distribution in this setting. Future research should optimize study designs by using more refined instruments and multidimensional assessments of relationship quality. Qualitative studies could explore population-specific conceptions of high-quality relationships to inform the choice of appropriate instruments. Additionally, including information on smoking, drinking, and diet may help to explore potential mediating pathways, and thus may lead to better explained variations in the outcomes. Lastly, longitudinal studies are needed to understand the temporal order and trajectories of relationship quality and health status. The majority of the population in our study was within normal BMI range and so it could possibly be difficult to see the association of relationship satisfaction and metabolic health, as this seems indicative that they were not from an area where metabolic health would be impacted by over-nutrition. Moreover, taking into consideration that average BMI was within the normal range, this may provide additional clarification as to why there was no observed mediation effect between depressive symptoms and waist circumference. One possible explanation for this discrepancy could be that BMI and waist circumference represent different aspects of body composition. BMI is a measure of overall body weight relative to height, while waist circumference is a measure of abdominal fat distribution. Therefore, future studies also need to be explored in a population that specifically includes obese Burkinabe people.

## Conclusion

In contrast to findings in high-income countries, this study discovered that higher levels of relationship satisfaction were associated with increased body weight among adults in Burkina Faso. However, longitudinal data on the correlations between relationship satisfaction and body weight are not yet available for this cohort and these results could be influenced by different beauty ideals or health literacy in Burkina Faso. Nevertheless, it is possible that relationship satisfaction alone may not have a unique association with body weight when considering factors like depressive symptoms and physical inactivity.

### Electronic supplementary material

Below is the link to the electronic supplementary material.

Supplementary Material 1

Supplementary Material 2

Supplementary Material 3

## Data Availability

The data that support the findings of this study are available from Centre de Recherche en Santé de Nouna Heidelberg Aging Study (CHAS) but are restricted for research use only. The datasets generated and/or analysed during the current study are not publicly available. Data are available upon reasonable request to Dr Guy Harling (g.harling@ucl.ac.uk).

## Abbreviations

BMI: Body Mass Index
CHAS: Centre de Recherche en Santé de Nouna Heidelberg Aging Study
CRSN: Centre de Recherche en Santé de Nouna
CSI-4: Couples Satisfaction Index 4
HbA1c: Glycated Hemoglobin A1c
HDSS: Health and Demographic Surveillance System
PHQ-9: Patient Health Questionnaire 9
WC: Waist circumference
