# Supplementary material for: Relationship satisfaction and metabolic health parameters: a cross-sectional study in Burkinabe population of older adults
Source: BMC Public Health. 2024 Mar 15;24:827. doi: 10.1186/s12889-024-17998-w (PMC10943782; doi:10.1186/s12889-024-17998-w)
Supplement: Supplementary file 1 — Supplementary Material 1 [file 12889_2024_17998_MOESM1_ESM.docx]

**Relationship satisfaction (CSI-4)**

**WC**

**Depressive symptoms (PHQ-9)**

a

b

c´

-0.111 (-0.152 – -0.071)^***^

-0.096 (-0.213 – 0.020)

0.114 (0.213 – 0.020)^*^

**Supplementary Figure 1.** Mediation model of depressive symptoms measured by PHQ-9 on relationship satisfaction measured by CSI-4 and WC. Data are presented as beta coefficient and 95% confidence intervals in parentheses and adjusted for model 4. CSI-4: Couples Satisfaction Index 4; PHQ-9: Patient Health Questionnaire 9; WC: Waist Circumference. The Monte Carlo mediation analysis test obtained with *medsem* commands showed insignificant results (β = 0.011, 95% CI: -0.002 – 0.026, p = 0.120), which suggests that no mediation effect was found. *** p<0.001, *p<0.05.
