# Supplementary material for: Relationship satisfaction and metabolic health parameters: a cross-sectional study in Burkinabe population of older adults
Source: BMC Public Health. 2024 Mar 15;24:827. doi: 10.1186/s12889-024-17998-w (PMC10943782; doi:10.1186/s12889-024-17998-w)
Supplement: Supplementary file 2 — Supplementary Material 2 [file 12889_2024_17998_MOESM2_ESM.docx]

Supplementary Table 1. Hierarchical multinomial logistic regression for BMI and central obesity (Ref: normal, *n* = 1458 for BMI and *n* = 1420 for WC)

| ***Outcome*** | ***Predictors*** | **Model 1** | | |  | **Model 2** | | |  | **Model 3** | | |  | **Model 4** | | |
| --- | --- | --- | --- | --- | --- | --- | --- | --- | --- | --- | --- | --- | --- | --- | --- | --- |
|  |  | ***OR*** | ***95% CI*** | ***P*** |  | ***OR*** | ***95% CI*** | ***P*** |  | ***OR*** | ***95% CI*** | ***P*** |  | ***OR*** | ***95% CI*** | ***P*** |
| **UW**  **(n = 317)** | **CSI-4** | 0.98 | 0.95 – 1.02 | 0.416 |  | 0.99 | 0.96 – 1.03 | 0.613 |  | 0.99 | 0.96 – 1.03 | 0.842 |  | 0.98 | 0.93 – 1.03 | 0.434 |
|  | **PHQ-9** |  |  |  |  |  |  |  |  | 1.05 | 1.01 – 1.09 | 0.011 |  | 1.05 | 1.01 – 1.09 | 0.011 |
|  | **Sitting h/week** |  |  |  |  |  |  |  |  | 1.00 | 0.99 – 1.01 | 0.416 |  | 1.00 | 0.99 – 1.01 | 0.416 |
|  | **CSI: Gender** |  |  |  |  |  |  |  |  |  |  |  |  | 1.04 | 0.96 – 1.11 | 0.320 |
|  | **CSI:Age** |  |  |  |  |  |  |  |  |  |  |  |  | 1.00 | 0.99 – 1.00 | 0.914 |
| **OW/Obese**  **(n = 446)** | **CSI-4** | **1.04** | **1.01 – 1.07** | **0.005** |  | 1.03 | 0.99 – 1.06 | 0.079 |  | 1.02 | 0.99 – 1.06 | 0.127 |  | 1.04 | 0.99 – 1.09 | 0.083 |
|  | **PHQ-9** |  |  |  |  |  |  |  |  | 0.98 | 0.94 – 1.01 | 0.169 |  | 0.97 | 0.94 – 1.01 | 0.176 |
|  | **Sitting h/week** |  |  |  |  |  |  |  |  | 1.01 | 1.00 – 1.02 | 0.011 |  | 1.01 | 1.00 – 1.02 | 0.011 |
|  | **CSI: Gender** |  |  |  |  |  |  |  |  |  |  |  |  | 0.97 | 0.91 – 1.04 | 0.380 |
|  | **CSI:Age** |  |  |  |  |  |  |  |  |  |  |  |  | 1.00 | 0.99 – 1.00 | 0.957 |
| **Central obesity ^a^**  **(n = 803)** | **CSI-4** | **1.04** | **1.01 – 1.07** | **0.015** |  | 1.02 | 0.99 – 1.05 | 0.212 |  | 1.01 | 0.99 – 1.05 | 0.267 |  | 1.04 | 0.99 – 1.10 | 0.078 |
|  | **PHQ-9** |  |  |  |  |  |  |  |  | 0.98 | 0.95 – 1.01 | 0.257 |  | 0.98 | 0.95 – 1.01 | 0.252 |
|  | **Sitting h/week** |  |  |  |  |  |  |  |  | 0.99 | 0.99 – 1.01 | 0.624 |  | 0.99 | 0.99 – 1.01 | 0.625 |
|  | **CSI: Gender** |  |  |  |  |  |  |  |  |  |  |  |  | 0.96 | 0.90 – 1.02 | 0.164 |
|  | **CSI:Age** |  |  |  |  |  |  |  |  |  |  |  |  | 1.00 | 0.99 – 1.00 | 0.980 |
| Model 1: adjusted for age, gender, and ethnicity.  Model 2: additionally adjusted for wealth and education.  Model 3: additionally adjusted for PHQ-9 and sitting time.  Model 4: additionally adjusted for interaction terms between CSI-4 and age/gender.  BMI: Body Mass Index; UW: underweight (< 18.5 kg/m^2^); normal (18.5 – 25.0 kg/m^2^); OW: overweight (25.0 – 30.0 kg/m^2^); Obese (>30.0 kg/m^2^).  ^a^ central obesity defined as a waist circumference ≥ 94 cm in men or ≥ 80 cm in women according to International Diabetes Federation (IDF)  Prevalence of underweight, normal, overweight (n = 346), and obese (n = 100) was 14.3%, 65.6%, 15.6%, and 4.5% respectively | | | | | | | | | | | | | | | | |
