# Supplementary material for: Relationship satisfaction and metabolic health parameters: a cross-sectional study in Burkinabe population of older adults
Source: BMC Public Health. 2024 Mar 15;24:827. doi: 10.1186/s12889-024-17998-w (PMC10943782; doi:10.1186/s12889-024-17998-w)
Supplement: Supplementary file 3 — Supplementary Material 3 [file 12889_2024_17998_MOESM3_ESM.docx]

Supplemental Table 2. Hierarchical multiple linear regression models for HbA1c.

| ***Predictors*** | **Model 1** | | |  | **Model 2** | | |  | **Model 3** | | |  | **Model 4** | | |
| --- | --- | --- | --- | --- | --- | --- | --- | --- | --- | --- | --- | --- | --- | --- | --- |
|  | ***β*** | ***95% CI*** | ***p*** |  | ***β*** | ***95% CI*** | ***p*** |  | ***β*** | ***95% CI*** | ***p*** |  | ***β*** | ***95% CI*** | ***p*** |
| **CSI-4** | 0.00 | -0.01 – 0.01 | 0.323 |  | -0.00 | -0.01 – 0.01 | 0.835 |  | -0.00 | -0.01 – 0.01 | 0.899 |  | 0.00 | -0.01 – 0.01 | 0.590 |
| **Age** | **0.00** | **0.00 – 0.01** | **0.001** |  | **0.01** | **0.00 – 0.01** | **<0.001** |  | **0.01** | **0.00 – 0.01** | **<0.001** |  | **0.01** | **0.00 – 0.01** | **<0.001** |
| **Female** | 0.01 | -0.03 – 0.06 | 0.617 |  | -0.01 | -0.06 – 0.04 | 0.678 |  | -0.01 | -0.06 – 0.03 | 0.596 |  | 0.06 | -0.10 – 0.22 | 0.447 |
| **Ethnicity:**  **Bwaba** | **-0.11** | **-0.17 – -0.06** | **<0.001** |  | **-0.09** | **-0.15 – -0.04** | **0.001** |  | **-0.09** | **-0.15 – -0.04** | **0.001** |  | **-0.09** | **-0.15 – -0.04** | **0.001** |
| **Ethnicity:**  **Mossi** | -0.05 | -0.12 – 0.02 | 0.187 |  | -0.06 | -0.13 – 0.10 | 0.096 |  | -0.06 | -0.13 – 0.11 | 0.102 |  | -0.06 | -0.13 – 0.10 | 0.094 |
| **Ethnicity:**  **Peulh** | **-0.15** | **-0.24 – -0.07** | **<0.001** |  | **-0.08** | **-0.17 – -0.00** | **0.045** |  | **-0.08** | **-0.17 – -0.00** | **0.045** |  | **-0.09** | **-0.17 – -0.00** | **0.043** |
| **Ethnicity:**  **Samo** | 0.08 | -0.02 – 0.17 | 0.118 |  | 0.04 | -0.05 – 0.13 | 0.365 |  | 0.04 | -0.05 – 0.13 | 0.365 |  | 0.04 | -0.05 – 0.13 | 0.369 |
| **Ethnicity:**  **Other** | 0.07 | -0.10 – 0.23 | 0.418 |  | -0.04 | 0.20 – 0.13 | 0.676 |  | -0.03 | 0.20 – 0.13 | 0.689 |  | -0.04 | 0.20 – 0.13 | 0.666 |
| **Wealth L2** |  |  |  |  | -0.02 | -0.10 – 0.05 | 0.509 |  | -0.02 | -0.10 – 0.05 | 0.572 |  | -0.02 | -0.10 – 0.05 | 0.580 |
| **Wealth L3** |  |  |  |  | 0.04 | -0.03 – 0.12 | 0.268 |  | 0.05 | -0.03 – 0.12 | 0.229 |  | 0.05 | -0.03 – 0.12 | 0.233 |
| **Wealth L4** |  |  |  |  | **0.08** | **0.01 – 0.15** | **0.046** |  | **0.08** | **0.01 – 0.16** | **0.037** |  | **0.08** | **0.00 – 0.15** | **0.038** |
| **Wealth L5** |  |  |  |  | **0.12** | **0.04 – 0.21** | **0.003** |  | **0.13** | **0.04 – 0.21** | **0.003** |  | **0.13** | **0.04 – 0.21** | **0.003** |
| **Years of study** |  |  |  |  | 0.00 | -0.01 – 0.01 | 0.602 |  | 0.00 | -0.01 – 0.01 | 0.652 |  | 0.00 | -0.01 – 0.01 | 0.657 |
| **Hypertension** |  |  |  |  | **0.10** | **0.05 – 0.15** | **<0.001** |  | **0.10** | **0.05 – 0.15** | **<0.001** |  | **0.10** | **0.05 – 0.15** | **<0.001** |
| **BMI** |  |  |  |  | **0.02** | **0.02 – 0.03** | **<0.001** |  | **0.02** | **0.02 – 0.03** | **<0.001** |  | **0.02** | **0.02 – 0.03** | **<0.001** |
| **PHQ-9** |  |  |  |  |  |  |  |  | 0.00 | -0.00 – 0.01 | 0.346 |  | 0.00 | -0.00 – 0.01 | 0.337 |
| **Sitting h/week** |  |  |  |  |  |  |  |  | 0.00 | -0.00 – 0.00 | 0.081 |  | 0.00 | -0.00 – 0.00 | 0.079 |
| **CSI:Gender** |  |  |  |  |  |  |  |  |  |  |  |  | -0.01 | -0.02 – 0.01 | 0.337 |
| **CSI:Age** |  |  |  |  |  |  |  |  |  |  |  |  | -0.00 | -0.00 – 0.00 | 0.837 |
| **R^2^/adj.R^2^** | 0.023/0.019 | | |  | 0.084/0.078 | | |  | 0.086/0.079 | | |  | 0.087/0.078 | | |
| Model 1: adjusted for age, gender, and ethnicity.  Model 2: additionally adjusted for wealth, education, hypertension, and BMI.  Model 3: additionally adjusted for PHQ-9 and sitting time.  Model 4: additionally adjusted for interaction terms between CSI-4 and age/gender.  β: beta-coefficient; SE: Standard Error; p: p-value; CSI-4: Couples Satisfaction Index 4; PHQ-9: Patient Health Questionnaire 9 | | | | | | | | | | | | | | | |
